# Selective internal radiation therapy of metastatic breast cancer to the liver: A meta-analysis

**DOI:** 10.3389/fonc.2022.887653

**Published:** 2022-11-24

**Authors:** Chenyu Liu, George Tadros, Quinn Smith, Linda Martinez, James Jeffries, Zhiyong Yu, Qian Yu

**Affiliations:** ^1^ School of Medicine, George Washington University, Washington DC, United States; ^2^ Department of Surgery, Cleveland Clinic Florida, Weston, FL, United States; ^3^ Kansas City University, College of Osteopathic Medicine, Kansas City, MO, United States; ^4^ School of Medicine, Ross University, Miramar, FL, United States; ^5^ Interventional Radiology, University of Chicago, Chicago, IL, United States; ^6^ Department of Breast Surgery, Shandong Cancer Hospital and Institute, Shandong First Medical University and Shandong Academy of Medical Sciences, Jinan, China

**Keywords:** breast cancer, liver metastases, yttrium, RECIST, interventional radiology, radioembolization

## Abstract

**Introduction:**

The aim of this study is to conduct a meta-analysis to assess the efficacy of yttrium-90 selective internal radiation therapy (SIRT) in treating patients with breast cancer with hepatic metastasis.

**Method:**

PubMed and The Cochrane Library were queried from establishment to January 2021. The following keywords were implemented: “breast”, “yttrium”, and “radioembolization”. The following variables and outcomes were collected: publication year, region, sample size, study design, presence of extrahepatic disease, tumor burden, infused radioactivity, breast cancer subtype, previous treatment, median survival time (MST), length of follow-up, adverse events, and radiographical response such as Response Evaluation Criteria in Solid Tumors (RECIST), modified RECIST (mRECIST), and Positron Emission Tomography Response Criteria in Solid Tumors (PERCIST).

**Results:**

A total of 24 studies from 14 institutions were included in the present meta-analysis. On the basis of the data from 412 patients, post-embolization MST was 9.8 [95% confidence interval (CI): 9.0–11.6] months. Patients with additional extrahepatic metastasis had a poorer survival rate compared with those with localized hepatic metastasis only (MST: 5.3 vs. 15 months, p < 0.0001). Patients with <25% liver tumor burden exhibited more promising survival than those with >25% (MST: 10.5 vs. 6.8 months, p < 0.0139). On the basis of RECIST, mRECIST, and PERCIST criteria, tumor response rate was 36% (95% CI: 26%–47%), 49% (95% CI: 34%–65%), and 47% (95% CI: 17%–78%), respectively, whereas tumor control rate was 85% (95% CI: 76%–93%), 73% (95% CI: 59%–85%), and 97% (95% CI: 91%–100%), respectively.

**Conclusion:**

On the basis of the available published evidence, SIRT is feasible and effective in treating patients with breast cancer with liver metastasis. Patients with lower hepatic tumor burden and without extrahepatic metastasis demonstrated more survival benefit. Future randomized controlled trials are warranted.

## Introduction

Breast cancer is the most common cancer globally. Currently, breast cancer affects approximately 12% of women globally (1). While the outcomes of localized primary breast cancer can be successfully eradicated by surgery with promising survival, the outcomes of metastatic breast cancer are abysmal. Liver metastases comprise half of all breast cancer malignancies, carrying with them an inauspicious prognosis and a scant 5-year survival of 8.5% (2). Medical treatment of metastatic breast cancer is directed by tumor subtype, such as hormonal therapy for estrogen receptor (ER)–positive subtypes and trastuzumab for Human epidermal growth factor receptor 2 HER2-positive subtypes. For hepatic metastasis, surgery, external beam radiation therapy (EBRT), and logoregional therapy such as chemoembolization and thermoablation are also effective in reducing tumor burden and prolonging survival (3). Despite a variety of treatment options, it is difficult to treat patients with large metastatic breast cancer to the liver recalcitrant to medical therapy and cannot tolerate surgery or EBRT. Selective internal radiation therapy (SIRT) with yttrium-90 (Y-90) has emerged as an effective treatment for unresectable hepatocellular carcinoma, cholangiocarcinoma, and secondary liver tumors such as colorectal cancer and uveal melanoma metastasis (4–7). In the last few decades, several single-center retrospective cohort studies reported its use in metastatic breast cancer to the liver (6–14). The aim of the present study is to investigate the safety and efficacy of SIRT in treating metastatic breast cancer through meta-analysis.

## Material and method

### Literature screening

This meta-analysis was performed in accordance with the Preferred Reporting Items for Systematic Review and Meta-analysis (PRISMA) guideline (15). PubMed and The Cochrane Library were searched from establishment to January 2021. The following keyword terms were used: “breast” AND (“radioembolization” OR “yttrium”) for PubMed; “breast”, “yttrium”, and “radioembolization” for The Cochrane Library.

**Figure 1:**
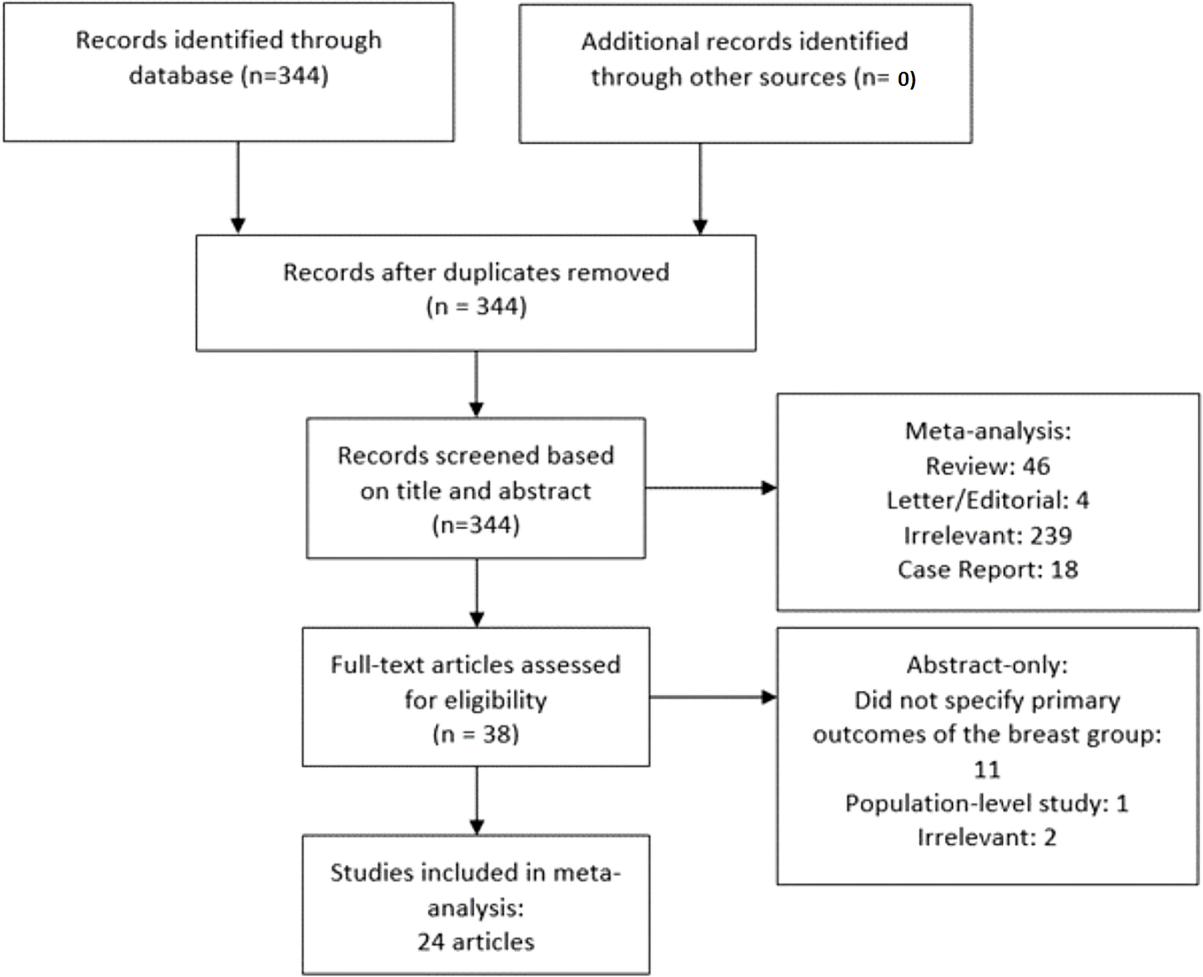
Flow diagram of literature screening.

The following inclusion criteria were adopted: a) patient was diagnosed with metastatic breast cancer to the liver and received SIRT; b) primary clinical outcomes including radiological response and/or survival rates were reported. A study was excluded if the following criteria were met: a) non-human studies; b) case report and study with sample size ≤5 patients; c) absence of original data (letter, editorial, commentary, and review); and d) population-level study. Endnote X8 (Clarivate Analytics, Philadelphia, PA, USA) was used to identify and remove duplicates. Articles were initially screened on the basis of titles, abstracts, and keywords, followed by a comprehensive review of full text of the remaining studies. A detailed screening process was depicted in **Figure 1**.

### Data acquisition

The primary endpoints of interest were median survival time (MST), overall survival (OS), and degree of radiographic response. The secondary endpoints were treatment-related adverse events and predictor analysis of OS [hazard ratio (HR)]. The following baseline characteristics were retrieved: publication year, region, sample size, study design, presence of extrahepatic disease, tumor burden, infused radioactivity, breast cancer subtype, previous treatment, MST, length of follow-up, adverse events, and follow-up length. Measures of radiographic response, classified as complete response (CR), partial response (PR), stable disease (SD), and progressive disease (PD), were extracted along with the imaging evaluation methods. Tumor response rate (TRR) was defined as the combined rates of CR and PR; tumor control rate (TCR) was defined as the combined rates of CR, PR, and SD. Subgroup analysis was performed on the basis of the radiological response criteria: Response Evaluation Criteria in Solid Tumors (RECIST), modified RECIST (mRECIST) and Positron Emission Tomography Response Criteria in Solid Tumors (PERCIST). Two authors retrieved the data independently. Any disagreement was resolved upon discussion.

The MST and OS at 6 months, 1 year, 2 years, and 3 years were extracted. Individual patient survival outcomes were retrieved from the survival curves of the original studies using GetGraph Digitizer v 2.26 (http://getdata-graph-digitizer.com/). For studies that did not label censored data (tick), only data of patients who died during follow-up were included, as the length of survival of patients who remained alive or lost-to-follow-up could not be extracted. OS rate was pooled using extracted individual survival outcomes on a Kaplan–Meier survival curve. MST and cumulative survival rates at 6 months, 1 year, 2 years, and 3 years were calculated. The quality of each study was assessed with the NIH Quality Assessment Tool for Observational Cohort and Cross-sectional Studies (**Supplementary Table 1**). For studies involving overlapping patient samples, only the largest cohort was included in the quantitative analysis for the survival outcomes (**Supplementary Table 2**).

### Statistical analysis

All quantitative analyses were performed with Stata 15.1 (STATA Corp., College Station, TX, USA). Meta-analysis was conducted with *-metan* and *-metaprop one* functions. The radiological response, HR of predictive analysis, and adverse events were pooled and reported in weighted means with 95% Wald confidence interval (CI). A random-effects model was adopted because of the cross-study heterogeneity. Publication bias was analyzed with the Egger’s test and by assessing asymmetries on the funnel plot. For studies sharing overlapping patient samples, only the study with the largest sample size was included in the quantitative analysis for the variable of interest. The log-rank test was implemented to compare survival outcomes between groups. P-value <0.05 was considered significant.

## Results

### Baseline characteristics of included studies

Among 344 initial search results, reviews (n = 46), letter/editorial (n = 4), case reports (n = 18), population level studies (n = 1), irrelevant (n = 240), and studies that did not specify primary outcomes for the breast metastasis subgroup (n = 11) were excluded, yielding 24 articles from 14 unique patient cohorts (**Figure 1**) **(** 8–14, 16–32). All studies were retrospective or non-comparative designs except that by Aarts et al. (8), which prospectively compared trans-arterial chemoembolization (TACE) and SIRT (interchangeable). The sample size of each study ranged from 16 to 81 patients. Mean radioactivity infused ranged from 1.6 to 2.1 GBq. Eleven and two studies implemented either resin or glass microspheres, respectively; five studies included patients underwent both; two studies did not specify the type of beads used. The distribution of breast cancer molecular subtype (ER, PR, HER2, and Triple negative breast cancer TNBC), prior treatment, concomitant systemic therapy, and follow-up length of each individual study were listed in **Tables 1**, **2**.

**Table 1:** Baseline characteristics of studies.

Study	Region	Design	Assessment criteria	Sample size	Extrahepatic disease	Type of microsphere	Activity infused(GBq)	Follow-up
**Aarts, 2020**	Netherlands	Prospective	RECIST	16	9/16	Resin	1.68 GBq (1.043–2.140)	6–8 weeks
Bagni, 2015*	Italy	prospective	PERCIST	17	10/17	Resin	1.8 ± 0.7 GBq	8 weeks
Cianni, 2012*	Italy	Retrospective	RECIST	52	24/52	Resin	Median: 1.9 GBq (0.33–2.71 GBq)	8 weeks
Cianni, 2010*	Italy	Retrospective	RECIST	Not listed due to repeated sample				
Bangash, 2007**	USA	Retrospective	RECIST	27	Not reported	Glass	1.70 GBq (2.05 GBq ± 1.06)	90 days
Gordon, 2014**	USA	Retrospective	PET/CA15-3	75	58	Glass	1.52 GBq (95% CI: 1.38–1.67 GBq)	Median: 1.4 months
Seyal, 2014**	USA	Retrospective	RECIST	34 lesions	Not reported	Resin	Not reported	Not reported
**Chang, 2018**	USA	Retrospective	RECIST	30	20/30	Resin: 46 Glass: 3	0.79 GBq Range: 0.18–1.82 GBq	Median: 9 months (range: 1–109 months)
**Stuart, 2008**	USA	Retrospective	RECIST	7	1	Resin	Mean: 1.29 GBq ± 0.37 (0.6 –1.95 GBq)	3 months
**Fendler, 2015†**	Germany	Retrospective	mPERCIST	81	54	Resin	1.6 (0.6) GBq	Not reported
**Haug, 2011†**	Germany	Retrospective	RECIST & WHO	58	38/58	Resin	1.774 ± 0.492 GBq	27.5 weeks (range: 13–60 weeks)
**Jakobs, 2008¶**	Germany	Retrospective	RECIST	30	17/30	Resin	1.9 GBq	4.2 months (range: 1.6–5.6 months).
**Jakobs, 2007¶**	Germany	Retrospective	RECIST	Not listed due to repeated sample				
**Paprottka, 2017**	Germany	Retrospective	RECIST	40/385	Not reported			
**Paprottka, 2011¶**	Germany	Retrospective		Not listed due to repeated sample				
**Pieper, 2016.5‡**	Germany	Retrospective	RECIST	44	44	Resin: 56/69 Glass: 13/69	1.35 (± 0.71)	Median: 121 days
**Pieper, 2016.7‡**	Germany	Retrospective	RECIST	21	Not listed due to repeated sample			
**Saxena, 2013**	Australia	Retrospective	RECIST	40	24	Resin	1.67 ± 0.36 GBq Range: 0.79–2.38 GBq	11.2 months (0.6–30.5 months)
**Barabasch, 2018**	Germany	Prospective	RECIST	14/36	21/36	Resin 34 Glass: 2	Left lobe: 0.69 GBq ± 0.18 (n = 9) Right lobe: 1.21 GBq ± 0.41 (n = 27)	4–6 weeks
**Coldwell, 2007**	USA	Retrospective	RECIST	44	43	Resin	Median: 2.1 GBq	Median: 14 months (1–42 months)
Deipolyi, 2020***	USA	Retrospective	mPERCIST	30	30	Resin: 24 Glass: 14	Resin: 22.7 ± 8.8 mCi Glass: 66.9 ± 42.5 mCi	51 ± 51 days (range: 1–243 days)
Deipolyi, 2018***	USA	Retrospective	RECIST	Not listed due to repeated sample				
**Davisson, 2020**	USA	Retrospective	RECIST	24	17	Resin: 19 Glass: 4 Mix: 1	Right lobe (n = 7): median 31.3 mCi Left lobe (n = 1): median 27.7 mCi Bilobar (n = 16): median 44.3 mCi	76.5 days (26–265 days)
**Xing, 2016**	USA	Retrospective	Not reported	Not reported				

*, Santa Maria Goretti Hospital, Via Guido Renin, Latina, Italy; **, Northwestern University, Chicago, IL, USA; ***, Memorial Sloan Kettering Cancer Center, NY, USA; ¶, Group 1, Ludwig-Maximilians-University of Munich, Munich, Germany; †, Group 2, Ludwig-Maximilians-University of Munich, Munich, Germany; ‡, University of Bonn, Sigmund-Freud-Strasse 25, Bonn, Germany. Response Evaluation Criteria in Solid Tumors (RECIST), modified RECIST (mRECIST), and Positron Emission Tomography Response Criteria in Solid Tumors (PERCIST).

**Table 2:** Baseline characteristics of studies continued.

Study	Breast cancer subtype	Prior treatment	Concomitant systemic therapy	Tumor response rate (%)	Tumor control rate (%)	CR	PR	SD	PD	Total	Survival time
**Aarts, 2020**	ER+: 8/15 PR+: 8/15 Her2+: 15/15 TNBC: 6/15	Surgery: 1/16 Systemic therapy: 3/16		62.5%	100.0%	0	10	6	0	16	12.6 months (95% CI: 10.23–15.0)
Bagni, 2015*	ER+: 15/17 PR+: 13/17	N/A	N/A	100.0%	100.0%	2	15	0	0	17	Not listed due to repeat samples
Cianni, 2012*	N/A	Surgery: 9/52 TACE: 2/52 Radio ablation: 11/52 All received systemic therapy	N/A	55.8%	90.4%	0	29	18	5	52	11.5 months
Cianni, 2010*	Not listed due to repeat samples										
Bangash, 2007**	N/A	RFA: 1/27 TACE: 1/27 Hepatic resection: 1/27 All received systemic therapy	N/A	39.1%	91.3%	0	9	12	2	23	Not listed due to repeat samples
Gordon, 2014**	N/A	None: 66 Resection: 5 RF ablation: 5 TACE: 1	N/A	35.3%	98.5%	0	24	43	1	68	6.6 months (95% CI: 5.0– 9.2 months)
Seyal, 2014**	Not reported	All received systemic therapy	N/A	51.9%	74.1%	0	14	6	7	27	Not reported
**Chang, 2018**	ER+: 21/30 PR+: 20/30 Her2+: 2/30	Surgery/Ablation: 1/30 All received systemic therapy	3/30	41.4%	48.3%	0	12	2	15	29	12.9 months (95% CI: 5.3–19.7 months)
**Stuart, 2008**	Not reported	All received systemic therapy	Not reported								
**Fendler, 2015†**	ER+: 60/81 (74%) PR+: 40/81 (49%) Her2+: 28/81 (35%)	Prior local treatment: 20/81 (25%) Surgery: 8/81 (10%) RFA: 9/81 (11%) TACE: 4/81 (5%) LITT: 1/81 (1%) Multiple: 2/81 (2%)	N/A	51.8%	100.0%	0	29	27	0	56	35 weeks
**Haug, 2011†**	ER+: 45/51 PR+: 37/50 Her2+: 23/48	Prior local hepatic therapy: 17/58 Mean # of prior systemic therapy: 3.1 ± 1.8	N/A	25.6%	88.4%	0	11	27	5	43	47 weeks
**Jakobs, 2008¶**	HER2+: 6/30	All received systemic therapy. Hormonal: 24/30	N/A	60.9%	95.7%	0	14	8	1	23	Mean: 9.6 months (3–45.1 months)
**Jakobs, 2007¶**	Not listed due to repeat samples										
**Paprottka, 2017**	Not reported	N/A	N/A								227 days
**Paprottka, 2011¶**	Not listed due to repeat samples										
**Pieper, 2016.5‡**	ER+/PR+: 20/44 ER+/PR-: 6/44 ER-/PR-: 1/44	Systemic therapy: 44/44 Prior liver resection: 1/44 Previous transarterial chemoembolization: 4/44	12/44	39.5%	81.6%	0	15	16	7	38	Median OS after first TARE: 184 days (range: 29–2,331 days)
**Pieper, 2016.7‡**	Not listed due to repeat samples										
**Saxena, 2013**	n/a	Prior liver resection: 6/40 All received systemic therapy	1/40	31.6%	71.1%	2	10	15	11	38	13.6 months with a 24-month survival of 39%
**Barabasch, 2018**	14 breast cancer Unspecified types	Not specified for BC subgroup	N/A	11.1%	91.7%	0	4	29	3	36	36 weeks (95% CI: 24, 48).
**Coldwell, 2007**	ER+: 31/44 HER2+: 12/44	Failed systemic therapy: 32/44 Trastuzumab: 10/44 None has received surgery before	N/A	75.0%	95.0%	7	23	8	2	40	PD: 3.6 months Rest of the patient: 14 months (1–42 months) 86% alive at 14 months
**Davisson, 2020**	ER+: 20/24 PR+: 12/24 HER2+: 2/24 TNBC: 3/24	Previous liver directed therapy: 1/24 Systemic therapy: 24/24	20/24	8.7%	60.9%	0	2	12	9	23	35.4 months
Deipolyi, 2020***	ER+: 24/30 PR+: 20/30 HER2+: 7/30 TNBC: 2/30	Greater than 3 lines of systemic therapy: 30/30	N/A	80.0%	100.0%	0	12	3	0	15	38.9 months
Deipolyi, 2018***	Not listed due to repeat samples										
**Xing, 2016**	Not reported										LSF (<10%): 17.0 months LSF (>10%): 10.0 months

ER+, estrogen receptor positive; PR+, progesterone receptor positive; Her2+, Her2 positive by fluorescence in situ hybridization (FISH), fluorescence in situ hybridization; TNBC, triple-negative breast cancer; TACE, trans-arterial chemoembolization; CP, complete response; PR, partial response; SD, stable disease; PD, progressive disease; NA, not available; LSF, lung-shunt fraction.

*, Santa Maria Goretti Hospital, Via Guido Renin, Latina, Italy; **, Northwestern University, Chicago, IL, USA; ***, Memorial Sloan Kettering Cancer Center, NY, USA; ¶, Group 1, Ludwig-Maximilians-University of Munich, Munich, Germany; †, Group 2, Ludwig-Maximilians-University of Munich, Munich, Germany; ‡, University of Bonn, Sigmund-Freud-Strasse 25, Bonn, Germany.

### Overall survival

**Figure 2:**
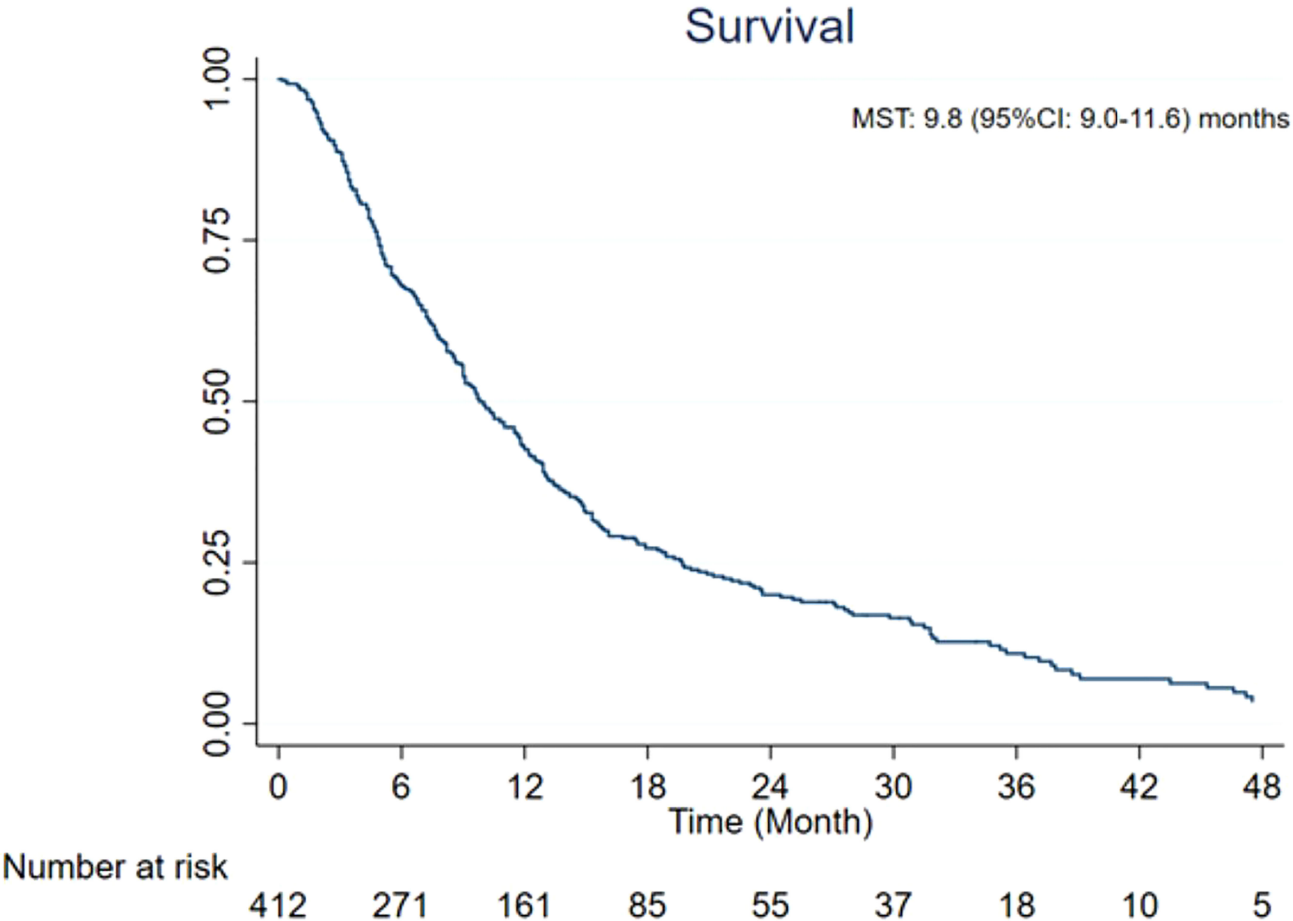
Overall survival from the time of radioembolization. CI, confidence interval; MST, median survival time.

**Figure 3:**
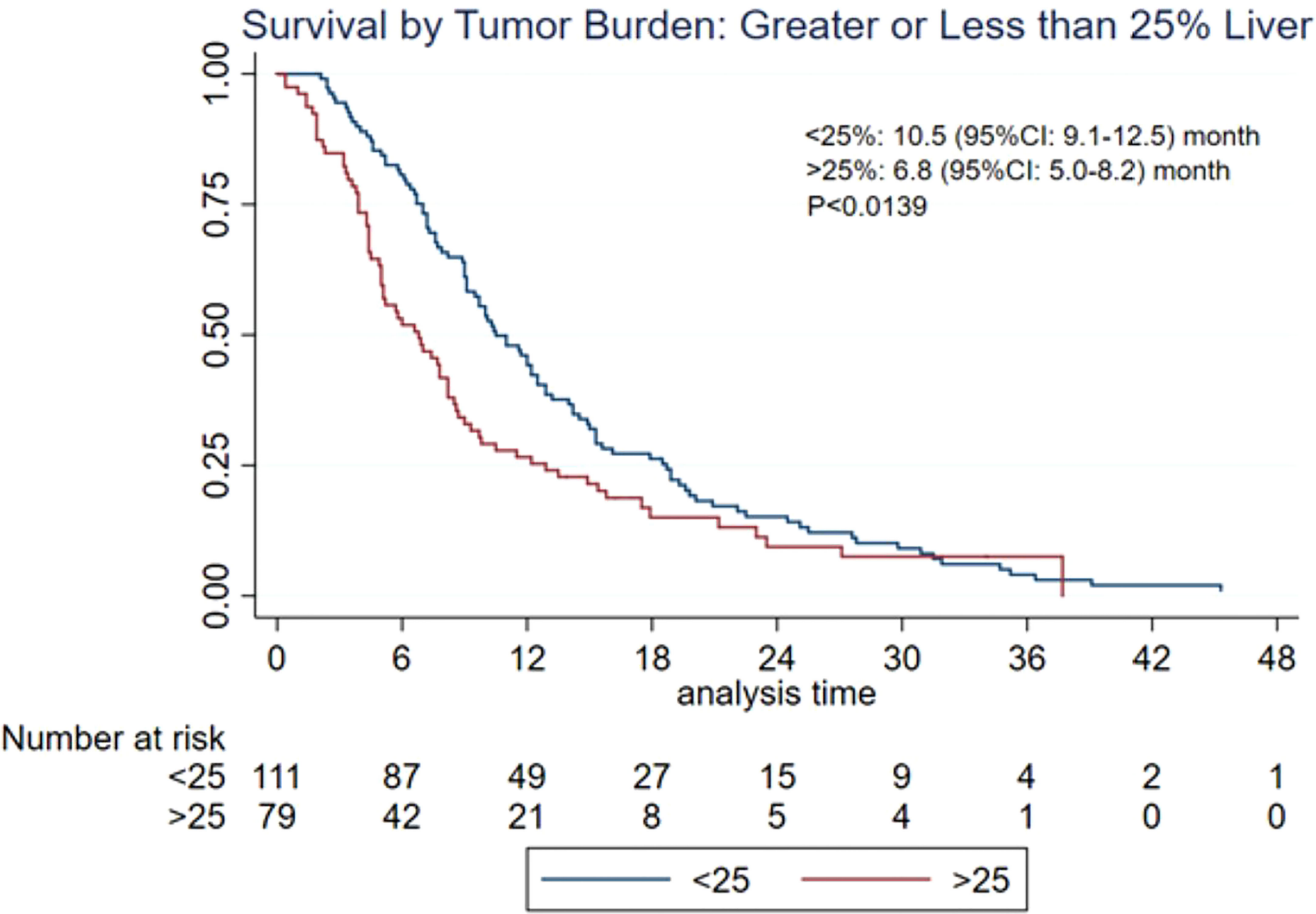
Overall survival based on hepatic tumor burden. CI, confidence interval; MST, median survival time.

**Figure 4:**
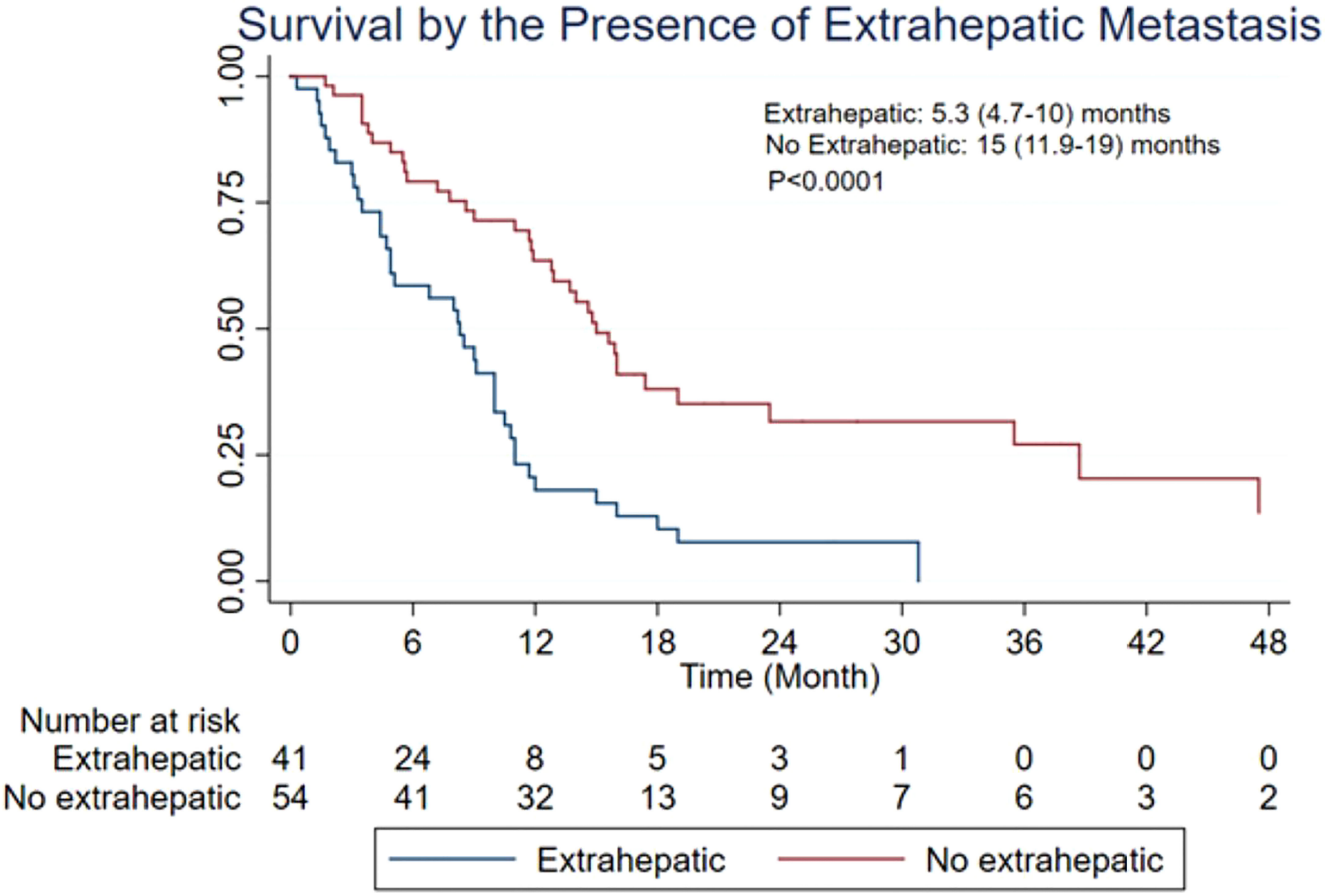
Overall survival based on the presence of extrahepatic metastatic disease. CI, confidence interval; MST, median survival time.

The MST of included studies ranged from 6.6 to 38.9 months (**Table 2**). On the basis of survival data from 412 patients, post-embolization MST was 9.8 (95% CI: 9.0–11.6) months (**Figure 2**). The cumulative OS rates at 6 months, 1 year, 2 years, and 3 years were 65.6% (95% CI: 60.8%–70.0%), 39.0% (95% CI: 34.3%–43.7%), 13.3% (95% CI: 10.3%–16.8%), and 4.4% (95% CI: 2.7%–6.6%), respectively. Patients with >25% hepatic metastatic burden had an MST of 6.8 months (95% CI: 5–8.2 months), compared with 10.5 months (95% CI: 9.1–12.5 months) of those with <25% burden (**Figure 3**, p < 0.0001). Patients with additional extrahepatic metastasis had a poorer survival rate compared with those with localized hepatic metastasis only (**Figure 4**; MST: 5.3 vs. 15 months, p < 0.0001).

### Radiological response

**Figure 5:**
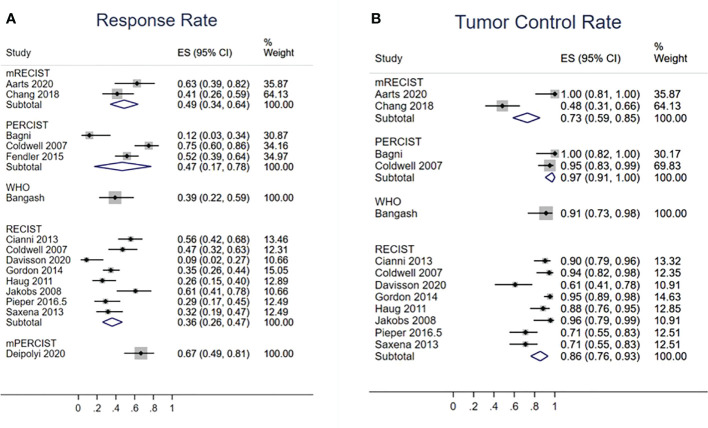
**(A)** Tumor response rate and **(B)** tumor control rate, stratified by imaging criteria.

Fourteen studies evaluated TRR (**Figure 5A**). According to the RECIST, mRECIST, and PERCIST criteria, TRRs were 36% (95% CI: 26%–47%), 49% (95% CI: 34%–65%), and 47% (95% CI: 17%–78%), respectively. Thirteen studies reported TCR (**Figure 5B**), which were 85% (95% CI: 76%–93%) by RECIST, 73% (95% CI: 59%–85%) by mRECIST, and 97% (95% CI: 91%–100%) by PERCIST. The funnel plot did not suggest asymmetry in evaluation of publication bias of RR (Egger test: p = 0.759) and TCR (Egger’s test: p = 0.173) based on the RECIST criteria (**Supplementary Figures 1A, B**).

### Adverse events

Post-embolization complication rates were pooled among 10 studies (**Table 3**; **Supplementary Table 3**). Cholecystitis occurred in seven of the 480 (1.5%) patients (≤grade 2). Sixteen of the 480 patients developed ulcers (3.0%), and nine of them were ≥grade 3 (2.1%). Two of the 480 patients had grade 3 pancreatitis (0.4%). The following biochemical toxicities (≥grade 3) were observed: elevated bilirubin (16 of 253, 6.3%), elevated aminotransferase (41 of 226, 18.1%), elevated alkaline phosphatase (4 of 91, 4.4%), leukocytosis (3 of 91, 3.3%), thrombocytopenia (0 of 16, 0%), and anemia (0 of 16, 0%).

**Table 3:** Adverse effects after yttrium-90 radioembolization sorted by complication type.

Adverse event	Rate (%)
**Cholecystitis/biliary complications**	7/480 (1.5%)
**Gastrointestinal ulcer**	16/480 (3.3%)
**Pancreatitis**	2/480 (0.4%)
**Hyperbilirubinemia**	16/253 (6.3%)
**Transaminitis**	41/226 (18.1%)
**Alkaline phosphatase elevation**	4/91 (4.4%)
**Thrombocytopenia**	0/16 (0%)
**Anemia**	0/16 (0%)
**Leukocytosis**	3/91(3.3%)

## Discussion

The present meta-analysis reviewed the available evidence and suggested that SIRT was feasible in treating patients with breast cancer with hepatic metastasis recalcitrant to other therapies with an overall post-embolization MST of 9.8 months. Patients with <25% and lack of extrahepatic disease showed a better response.

SIRT implements resin or glass microspheres embedded with radioactive isotope yttrium into tumors. Each bead exerts radiotherapy directly to adjacent tissues with approximately 2.5-mm penetration (33). Meanwhile, these beads also deprive tumors from arterial blood supply similar to TACE and bland Transarterial Embolization (TAE), leading to tissue ischemia and tumor necrosis. Although more than 70% blood supply of normal liver parenchyma derives from the portal venous system, hepatic malignancy is mainly supported by arteries. Tumor localization using transarterial microcatheters ensures SIRT’s selective delivery, spares normal tissue, and preserves liver functional reserve. Using SIRT segmentecotomy, >190 Gy of radiation dosage can be selectively delivered to a tumor-containing hepatic segment to achieve complete necrosis (34, 35). Because of its treatment precision, SIRT has been implemented for patients with Hepatocellular Carcinoma (HCC) who cannot tolerate sorafenib’s adverse effects. While initially considered as a palliative treatment for advanced stage HCC, SIRT has gradually been recognized for its curative role and is now an option for early and very early stage HCC according to the most recent Barcelona Clinic Liver Cancer guideline (36). The present study echoes evidence of SIRT on HCC by showing that SIRT can be a feasible option as a salvage treatment for metastatic breast cancer.

Available evidence of SIRT in breast cancer with hepatic metastasis mainly focused on tumors that are unresectable, recalcitrant to systemic treatment, and/or high liver disease burden. The 5-year survival rate of patients with metastatic breast cancer to the liver was 10% after systemic therapy (37), whereas the 5-year survival rate was 54% for patients with resectable disease. For unresectable breast cancer liver metastases, percutaneous ablative therapy using radiofrequency, laser, and microwave ablation are also feasible, with an MST of 10.9–54 months. The 5-year survival rate was 27%–35%, whereas local tumor progression was 2.9%–9.5% (38). Nonetheless, the seemingly more favorable outcomes associated with surgical and ablative treatment could be attributed to milder tumor burden compared with patients subjected to embolotherapy, which are often too large for resection and ablation. For breast cancer with heavy liver metastasis burden, transarterial chemoembolization, a treatment for BCLC stage B liver cancer and large colorectal metastasis, has been utilized with a promising MST from 7.3 to 47 months (39). In treatment of HCC, however, SIRT has gradually gained increasing popularity in the last decade. More pieces of evidence also became available supporting its use in metastatic cancer to the liver, such as colorectal metastasis and melanoma (7, 40). With both TACE and SIRT as the available options, comparative studies showed superior survival in among patients treated with the former (41, 42). As for breast cancer with liver metastasis, only Chang et al. compared TACE and SIRT in treating liver metastatic breast cancer, suggesting a longer MST (4.9 vs. 12.9 months) and fewer adverse events of SIRT (71% vs. 44%) (9). Further randomized controlled trials are warranted to validate these findings.

Compared with SIRT, EBRT has been historically more commonly used for unresectable hepatic metastasis. For breast cancer metastasized to the liver, stereotactic body radiation therapy (SBRT) offers a 1-year OS rate of 21%–85% (43–45). The 1- and 2-year local control rates, as measured by PERCIST criteria, were 100% and 80%, respectively (44). Compared with SIRT, EBRT of the liver faces the challenges of respiratory motion, requirement of fiducial marker, increased radiation to the normal parenchyma, multiple treatment sessions, limited dose delivery in the setting of large tumors, etc. (46, 47). While the comparative studies between EBRT and SIRT were lacking in the setting of breast cancer metastasis to the liver, a previous study on cholangiocarcinoma showed a seemingly longer survival of SIRT over EBRT as the first-line therapy (MST of 36 vs. 11 months) (48), highlighting SIRT’s effectiveness in reducing hepatic tumor burden as a novel therapy compared with the more traditional EBRT approach.

On the basis of the present study, the pooled TRR and TCR were 36%–49% and 73%–97%, respectively. These findings are similar to previously reported 22%–81% and 78%–96% in the primary liver cancer such as cholangiocarcinoma (48). Radiological response is a well-known predictor of survival after SIRT of HCC and colorectal liver metastasis (49, 50). According to Saxena et al., the radiological response also correlates with survival of patients with breast cancer with liver metastasis after SIRT (14). Among a variety of imaging criteria, RECIST and WHO criteria were developed first to characterize tumor response toward treatment by measuring uni- and bi-dimensional measurements in the liver cancer. Because anti-cancer effects can manifest as necrosis while maintaining a stable size, mRECIST is established to take this aspect into consideration (51). PERCIST, by contrast, can further evaluate the functionality of tumor by measuring glucose uptake (52). Although all these response criteria have been implemented in evaluating SIRT of the primary and secondary liver cancers, PERCIST and mRECIST have recently gained increasing popularity due to the accurate predictability of survival outcomes in hepatocellular carcinoma (53, 54). For metastatic breast cancer to the liver, the ideal radiological response criteria for survival prediction remain to be determined by the future comparative studies.

Whereas the included individual studies are heterogeneous in terms of patient population and disease burden, the present meta-analysis demonstrated that that the lower tumor burden and the absence of extrahepatic disease were associated with improved survival. Other reported factors include adjuvant chemotherapy, eastern cooperative oncology group performance status (ECOG) status, tumor vascularity, estrogen receptor status, baseline serum bilirubin and transaminase level, and [¹⁸F]Fluorodeoxyglucose (18F-FDG) standardized uptake value (13, 14, 21, 23–25). Some of these trends were consistent with the previous literature focusing on other treatment modalities in metastatic breast cancer and SIRT in HCC or colorectal cancer (55, 56). These findings advocate the early consideration of SIRT in patients with lower disease burden to achieve a more promising survival. The recent LEGACY study showed that SIRT could achieve 3-year OS rate of 87% for patients at early-stage HCC with ECOG 0-1 and unresectable tumor of up to 8 cm with 21% bridged to transplant or resection. The use of SIRT in breast cancer with limited hepatic metastasis burden is to be validated by the future studies.

In terms of safety, SIRT of breast cancer with liver metastasis is well tolerated without procedure-related death. One of the most serious side effects associated with SIRT, i.e., radiation-induced liver disease (RILD), did not occur among the included studies. On the basis of the evidence on HCC and colorectal metastasis, RILD typically has a reported incidence rate of less than 10% (57). The incidence rate of major gastrointestinal complications such as ulcer, cholecystitis, and pancreatitis were less than 3%. Careful angioanatomical planning and techniques such as coiling non-target vessels could decrease these risks. Radiation pneumonitis was not noted in the present study, which is rare nowadays due to measurement of lung-shunting fractions using dosimetry (58). Furthermore, the risk of retroperitoneal hematoma during femoral approach is significantly decreased with the use of closure devices. Alternative transradial approach has also gained popularity in SIRT, avoiding major vascular complications such as hematoma, pseudoaneurysm, and fistula formation.

The present meta-analysis should be interpreted with several caveats. First, the included studies were heterogeneous. Patients with different breast cancer subtypes, previous cancer therapy, concurrent chemotherapy, and disease burden were included. Stratification based on these variables is technically implausible with reported study-level outcomes. Second, the efficacy of SIR could be undermined because it is used as salvage treatment after patients failed multiple lines of treatments. Even patients with terminal disease and extra hepatic burden were included. The baseline survival of such patient population should be poorer than the survival rates reported in the literature, calculated from the diagnosis of hepatic breast metastasis. Third, radioembolization techniques were not specified. Whether superselective segmentectomy or whole liver Embolization was adopted would affect procedural safety profile. Furthermore, breast cancer with liver metastasis can be either hypo- or hypervascular (59). Hypervascular lesions could lead to higher radioembolization bead deposit and is more responsive toward SIRT, which was not analyzed in the present study. Last but not the least, most of the included studies were retrospective in design, categorized as level IV evidence. Future randomized controlled trial studies enrolling patients with a more homogenous baseline characteristics are warranted.

## Conclusion

SIRT is a feasible and effective treatment for breast cancer with liver metastasis. Patients with a low liver tumor burden and the lack of extrahepatic metastasis are more likely to convey favorable survival. Tumor responded toward SIRT on radiological follow-up evaluation. No life-threatening adverse effect occurred. However, the included patient population was heterogeneous in treatment history and disease severity, limiting the ability to draw broad conclusions. Given the non-comparative nature of most studies, future prospective and multicenter randomized controlled trials are warranted to determine the comparative efficacy of SIRT versus the other treatment approaches.

## Data availability statement

The original contributions presented in the study are included in the article/**Supplementary Material**. Further inquiries can be directed to the corresponding author.

## Author contributions

CL: Manuscript writing, data collection, statistical analysis GT: Manuscript writing, data collection QS: Data collection LM: Data collection JJ: Data collection ZY: Manuscript revision, guarantor QY: Manuscript writing/revision, statistical analysis, conceptualization, guarantor. All authors contributed to the article and approved the submitted version.

## Funding

This study is funded by grants from the Natural Science Foundation of Shandong Province (ZR2019MH109).

## Conflict of interest

The authors declare that the research was conducted in the absence of any commercial or financial relationships that could be construed as a potential conflict of interest.

## Publisher’s note

All claims expressed in this article are solely those of the authors and do not necessarily represent those of their affiliated organizations, or those of the publisher, the editors and the reviewers. Any product that may be evaluated in this article, or claim that may be made by its manufacturer, is not guaranteed or endorsed by the publisher.
