# Supplementary material for: Selective internal radiation therapy of metastatic breast cancer to the liver: A meta-analysis
Source: Front Oncol. 2022 Nov 24;12:887653. doi: 10.3389/fonc.2022.887653 (PMC9729947; doi:10.3389/fonc.2022.887653)
Supplement: Supplementary file 1 [file DataSheet_1.docx]

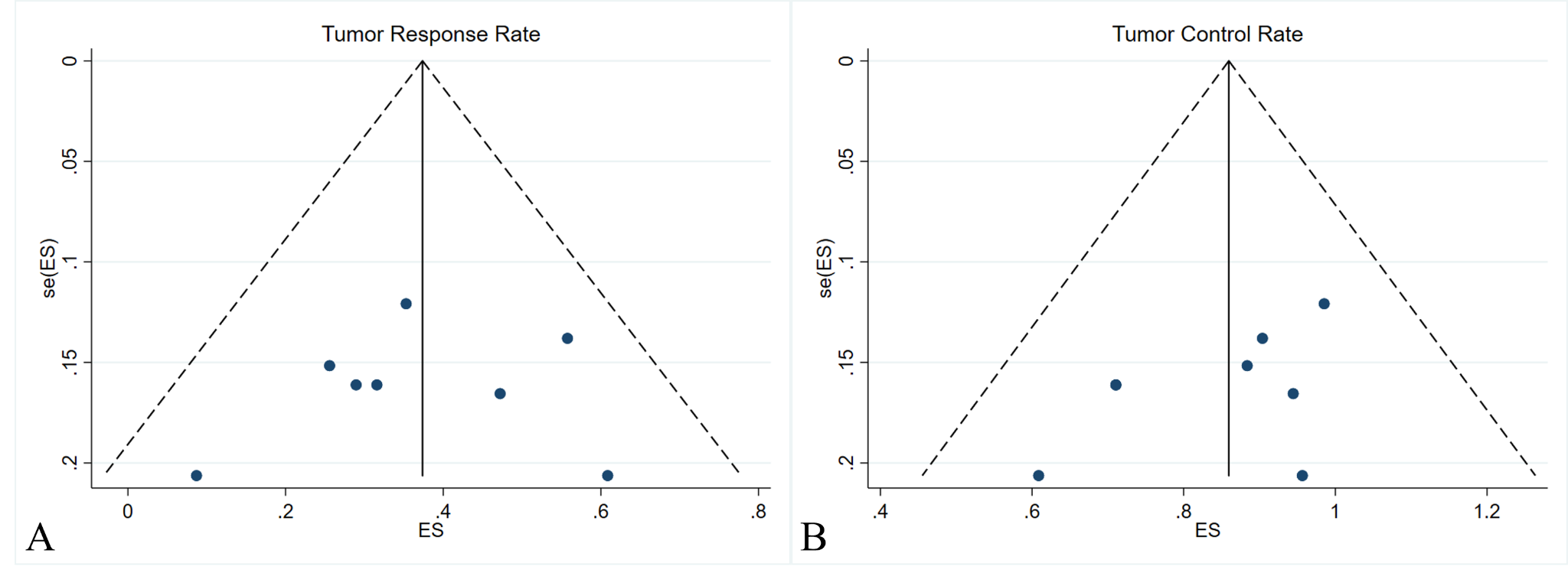


**Supplement Figure 1**: Funnel plot A) Tumor response rate and B) Tumor control rate

| Study | 1 | 2 | 3 | 4 | 5 | 6 | 7 | 8 | 9 | 10 | 11 | 12 | 13 | 14 |
| --- | --- | --- | --- | --- | --- | --- | --- | --- | --- | --- | --- | --- | --- | --- |
| Aarts 2020 | Y | Y | Y | Y,Y | Y | Y | Y | Y | Y | Y | Y | N | Y | N |
| Bagni 2015 | Y | Y | Y | Y,Y | Y | Y | Y | Y | Y | Y | Y | N | Y | N |
| Cianni 2012 | Y | Y | Y | Y,Y | Y | Y | Y | Y | Y | Y | Y | N | Y | N |
| Cianni 2010 | Y | Y | Y | Y,Y | Y | Y | Y | Y | Y | Y | Y | N | Y | N |
| Bangash 2007 | Y | Y | Y | Y,Y | Y | Y | Y | Y | Y | Y | Y | N | Y | N |
| Gordon 2014 | Y | Y | Y | Y,Y | Y | Y | Y | Y | Y | Y | Y | N | Y | N |
| Seyal 2014 | Y | Y | Y | Y,Y | Y | y | y | Y | Y | Y | Y | N | Y | N |
| Chang 2018 | Y | Y | Y | Y,Y | Y | Y | Y | Y | Y | Y | Y | N | Y | N |
| Stuart 2008 | Y | Y | Y | Y,Y | Y | Y | Y | Y | Y | Y | Y | N | Y | N |
| Fendler 2015 | Y | Y | Y | Y,Y | Y | Y | Y | Y | Y | Y | Y | N | Y | N |
| Haug 2011 | Y | Y | Y | Y,Y | Y | Y | Y | Y | Y | Y | Y | N | Y | N |
| Jakobs 2008 | Y | Y | Y | Y,Y | Y | Y | Y | Y | Y | Y | Y | N | N | N |
| Jakobs 2007 | Y | Y | Y | Y,Y | Y | Y | Y | Y | Y | Y | Y | N | Y | N |
| Paprottka 2017 | Y | Y | Y | Y,Y | Y | Y | Y | Y | Y | Y | Y | N | Y | N |
| Paprottka 2011 | Y | Y | Y | Y,Y | Y | Y | Y | Y | Y | Y | Y | N | Y | N |
| Pieper 2016.5 | Y | Y | Y | Y,Y | Y | Y | Y | Y | Y | Y | Y | N | Y | N |
| Pieper 2016.7 | Y | Y | Y | Y,Y | Y | Y | Y | Y | Y | Y | Y | N | Y | N |
| Saxena 2013 | Y | Y | Y | Y,Y | Y | Y | Y | Y | Y | Y | Y | N | Y | N |
| Barabasch 2018 | Y | Y | Y | Y,Y | Y | Y | Y | Y | Y | Y | Y | N | Y | N |
| Coldwell 2007 | Y | Y | Y | Y,Y | Y | Y | Y | Y | Y | Y | Y | N | Y | N |
| Deipolyi 2020 | Y | Y | Y | Y,Y | Y | Y | Y | Y | Y | Y | Y | N | Y | N |
| Deipolyi 2018 | Y | Y | Y | Y,Y | Y | Y | Y | Y | Y | Y | Y | N | Y | N |
| Davisson 2020 | Y | Y | Y | Y,Y | Y | Y | Y | Y | Y | Y | Y | N | Y | N |
| Xing 2016 | Y | Y | Y | Y,Y | Y | Y | Y | Y | Y | N | Y | N | Y | N |

**Supplement table 1**: Label: Y (yes), N (no). 1. Was the research question or objective in this paper clearly stated? 2. Was the study population clearly specified and defined? 3. Was the participation rate of eligible persons at least 50%? 4. Were all the subjects selected or recruited from the same or similar populations (including the same time period)? Were inclusion and exclusion criteria for being in the study prespecified and applied uniformly to all participants? 5. Was a sample size justification, power description, or variance and effect estimates provided? 6. For the analyses in this paper, were the exposure(s) of interest measured prior to the outcome(s) being measured? 7. Was the timeframe sufficient so that one could reasonably expect to see an association between exposure and outcome if it existed? 8. For exposures that can vary in amount or level, did the study examine different levels of the exposure as related to the outcome (e.g., categories of exposure, or exposure measured as continuous variable)? 9. Were the exposure measures (independent variables) clearly defined, valid, reliable, and implemented consistently across all study participants? 10. Was the exposure(s) assessed more than once over time? 11. Were the outcome measures (dependent

variables) clearly defined, valid, reliable, and implemented consistently across all study participants? 12. Were the outcome assessors blinded to

the exposure status of participants? 13. Was loss to follow-up after baseline 20% or less? 14. Were key potential confounding variables measured and adjusted statistically for their impact on the relationship between exposure(s) and outcome(s)?

| **Study/year** | **Overall Survival** | **Hepatic Burden** | **Intra- vs Extra-hepatic** |
| --- | --- | --- | --- |
| **Aarts 2020** | NR | - | - |
| **Bagni 2015*** | Overlapping Sample Size | - | Y |
| **Cianni 2012*** | Y | Y | - |
| **Cianni 2010*** | Overlapping Sample Size | - | - |
| **Bangash 2007**** | NR | - | - |
| **Gordon 2014**** | Y | Y | - |
| **Seyal 2014**** | NR | - | - |
| **Chang 2018†** | Y | - | - |
| **Stuart 2008†** | Y | - | - |
| **Fendler 2015 ¶¶** | Y | Y | - |
| **Haug 2011 ¶¶** | Overlapping Sample Size | - | - |
| **Jakobs 2008 ¶** | Y | - | Y |
| **Jakobs 2007 ¶** | Overlapping Sample Size | - | - |
| **Paprottka 2017** | Y | - | - |
| **Paprottka 2011 ¶** | NR | - | - |
| **Pieper 2016.5 ‡** | NR | - | - |
| **Pieper 2016.7‡** | Y | - | Y |
| **Saxena 2013** | Y | - | - |
| **Barabasch 2018** | NR | - | - |
| **Coldwell 2007** | NR | - | - |
| **Deipolyi 2020** | Y | - | - |
| **Deipolyi 2018** | Overlapping Sample Size | - | - |
| **Davisson 2020** | Y | - | Y |
| **Xing 2016** | NR | - | - |

**Supplement Table 2**: Studies included of survival analysis. NR: not reported. Y: Yes and included.

| **Studies** | **Sample Size** | **Hyperbilirubinemia** | **Transaminitis** | **Alkaline Phosphatase Elevation** | **Leukocytosis** | **Thombocytopenia** | **Anemia** | **Cholecystitis** | **Gastrointestinal Ulcer** | **Pancreatitis** |
| --- | --- | --- | --- | --- | --- | --- | --- | --- | --- | --- |
| Aarts | 16 | 0 | 0 | 0 | 0 | 0 | 0 | 0 | 5 | 0 |
| Bagni | 17 | - | - | - | - | - | - | 2 | 4 | 0 |
| Cianni | 52 | - | - | - | - | - | - | 2 | 4 | 0 |
| Bangash | 27 | 3 | - | - | - | - | - | 0 | 0 | 0 |
| Gordon | 75 | 4 | 6 | 4 | 3 | - | - | 0 | 0 | 0 |
| Chang | 30 | - | - | - | - | - | - | 0 | 0 | 0 |
| Coldwell | 44 | - | - | - | - | - | - | 0 | 2 | 0 |
| Davisson | 24 | 2 | 2 | - | - | - | - | 0 | 0 | 0 |
| Fendler | 81 | 5 | 31 | - | - | - | - | 0 | 0 | 2 |
| Jakobs | 30 | 2 | 2 | - | - | - | - | 0 | 0 | 0 |
| Pieper | 44 | - | - | - | - | - | - | 1 | 1 | 0 |
| Saxena | 40 | - | - | - | - | - | - | 2 | 0 | 0 |

**Supplement Table 3**: Complications by individual study.
